# Uncovering the secret weapons of an invasive plant: The endophytic microbes of *Anthemis cotula*

**DOI:** 10.1016/j.heliyon.2024.e29778

**Published:** 2024-04-19

**Authors:** Iqra Bashir, Aadil Farooq War, Iflah Rafiq, Zafar A. Reshi, Irfan Rashid, Yogesh S. Shouche

**Affiliations:** a Department of Botany, University of Kashmir, Srinagar, 190006, Jammu and Kashmir, India; b Azim PremJi University Bengaluru, Karnataka, India

**Keywords:** *Anthemis cotula,* plant invasion, Diversity, Biocontrol, Endophytes

## Abstract

Understanding plant-microbe interaction can be useful in identifying the microbial drivers of plant invasions. It is in this context that we explored the diversity of endophytic microbes from leaves of *Anthemis cotula*, an annual plant that is highly invasive in Kashmir Himalaya. We also tried to establish the role of endophytes in the invasiveness of this alien species. We collected and processed leaf samples from three populations at three different sites. A total of 902 endophytic isolates belonging to 4 bacterial and 2 fungal phyla were recovered that belonged to 27 bacterial and 14 fungal genera. Firmicutes (29.1%), Proteobacteria (24.1%), Ascomycota (22.8%) and Actinobacteria (19%) were dominant across all samples. Plant growth promoting traits, such as Ammonia production, Indole Acetic Acid (IAA) production, Phosphate solubilization and biocontrol activity of these endophytes were also studied and most of the isolates (74.68%) were positive for ammonia production. IAA production, phosphate solubilization and biocontrol activity was present in 39.24%, 36.70% and 20.26% isolates, respectively. Furthermore, *Botrytis cinerea,* a pathogen of *A. cotula* in its native range, though present in Kashmir Himalaya does not affect *A. cotula* probably due to the presence of leaf endophytic microbial antagonists. Our results highlight that the beneficial plant growth promoting interactions and enemy suppression by leaf endophytes of *A. cotula*, may be contributing to its survival and invasion in the Kashmir Himalaya.

## 1 Introduction

Successful invasions by non-native species in the introduced range is an outcome of many biotic and abiotic factors, such as propagule pressure [1], climate matching between the native and introduced [2], escape from enemies [3], higher competitive ability of invaders [4], release of novel chemicals by the invasive species [5] and mutualistic interactions [6]. Mutualisms have been shown to play an important role in the success of some alien plant species by improving their competitive ability [[7], [8], [9]]. Plants, including invasive species, are inhabited by a diverse array of mutualistic endophytic symbionts, including mycorrhizae, bacterial, and fungal endophytes in the inner tissues of various organs without displaying any negative impacts on the host plant [10]. Endophytes colonize all plant parts, such as roots, corms, stems, leaves, and floral organs [11,12] and the dynamic environment of the plant organs affects the structure and composition of endophytes that colonize different tissues [13,14].

While the role of endophytes in crop plants has been studied extensively, their involvement in invasiveness of alien species is still in its infancy. However, some recent studies have demonstrated the role of endophytes in increasing the competitiveness [9], growth [15], and protection against herbivores [16] in several invasive alien plant species. In particular, endophytes in the leaves of invasive plants bring about changes in leaf functional traits and tissue quality, which in turn, indirectly affect the herbivorous insects [17], chiefly through the production of secondary compounds which cause feeding deterrence or reduce insect growth due to toxicity and influence survival and oviposition [16]. However, some endophytes are reported to promote pathogen development [18] and such interactions could be exploited for integrated management of invasive plant species [19].

Keeping in the view the above-mentioned roles of endophytes, we explored the diversity of culturable leaf endophytes of *Anthemis cotula* (stinking chamomile), characterized them for their plant growth promoting activities and also tested them for their protection to the *A. cotula* against a common pathogenic fungus and a native fungal enemy. We choose *A. cotula* for two reasons: one that it is very widespread and abundant in Kashmir Himalaya [20] and second, its leaves emit pungent smell because of which it is commonly called as ‘stinking chamomile’ [21]. While studying the role of morpho-physiological [22], and reproductive [23] traits in the invasiveness of this species it has emerged that the leachate of pungent smelling leaves of *A. cotula* show allelopathic activity [23,24], thus making leaves as an organ of interest to us for studying the diversity and role of endophytes. We focussed on three aspects: (1) Diversity of bacterial and fungal endophytes in the leaves of *A. cotula,* (2) Plant growth promoting activities of endophytes, such as Indole acetic acid (IAA) production, phosphate solubilization, ammonia production, and (3) Biocontrol activity of endophytes against two phytopathogens, namely *Fusarium oxysporum* (a common soil pathogen) and *Botrytis cinerea* (enemy of *A. cotula* in its native range).

## 2 Materials and methods

### 2.1 Sample collection and endophyte isolation

**Fig. 1:**
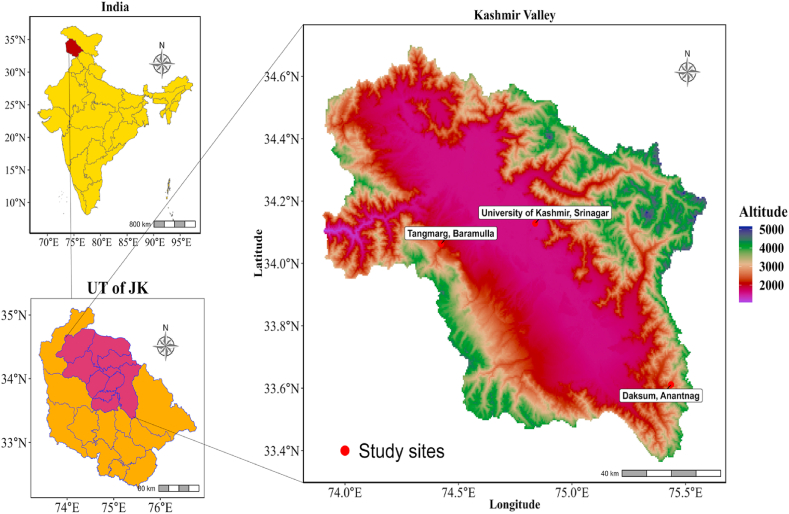
Map showing study area and sampling sites.

Leaf samples of *A. cotula* were collected in the month of May–June from three different sites in Kashmir valley that differed in altitude. Geographic location and altitude of each site are given in Table 1 and shown in the map also (Fig. 1).

**Table 1:** Geocoordinates of sampling sites.

S. No	Site	Latitude	Longitude	Altitude
01	Hazratbal, Srinagar	34° 7′44.42″N	74°50′32.57″E	1540 m
02	Tangmarg, Baramulla	34° 3′44.37″N	74°26′22.63″E	2000 m
03	Daksum, Anantnag	33°36′36.75″N	75°26′6.23″E	2345 m

Healthy leaf samples from 18 to 20 plants from each population were randomly collected from each site and each sample was put in individual sterile zip lock bags and stored at −20 °C, until further analysis. 100 leaves (five leaves per plant) from each sample were first washed with tap water to remove any attached debris followed by their surface sterilization as follows: 70% EtOH (3 min), 1.5%NaOCl (1 min), double distilled water washing (3 times). Surface sterilization was verified by imprinting surface-sterilized leaf samples and conducting spread plating of the final wash on nutrient agar plates to detect any microbial growth. Samples were dried aseptically using filter paper and then cut into smaller fragments with sterile surgical blade. Endophytes were first isolated following fragmentation technique, for which we analysed 200 segments (in triplicates). For fungal isolation, segments were placed on potato dextrose agar (PDA) and yeast malt agar (YMA) media plates supplemented with streptomycin (260 mg/L). However, for bacterial isolation, segments were placed on nutrient agar (NA) and Tryptic Soya Agar (TSA) plates supplemented with cycloheximide (50 μg/mL). Besides, serial dilution technique was also followed for isolation in which 1 gm of leaf was macerated in autoclaved motor pestle containing 0.9% saline solution. Then, 1 ml supernatant of the macerated samples was serial diluted (10^−1^-10^−7^) and 1 ml of each dilution was spread-plated on NA and PDA media plates separately. The plates were incubated for 14 days at 28 °C. Once the bacterial and fungal colonies appeared on the plates, each of them was 2–3 times sub-cultured on fresh media to obtain the pure cultures. The pure cultures were then preserved at −80 °C in 20% glycerol stock solutions for further characterization.

### 2.2 Molecular identification of obtained isolates

Genomic DNA from bacterial and fungal isolates was obtained using Wizard® Genomic DNA Purification Kit (Promega USA) and HiPurA® Fungal DNA Purification Kit, respectively following the manufacturers’ protocol. The identification was done by partial sequencing of 16S rRNA region of bacteria using universal 16S rDNA primer 27 F/1492 R (5ʹAGAGTTTGATCCTGGCTCAG/5ʹGGTTACCTTGTTACGACTT) (Sigma-Aldrich, India) and ITS region of fungi using the universal primers ITS1/ITS4 (5′-TCC GTAGTGAACCTGCGG-3′/5′-TCCTCCGCTTAT TGATATGC-3′) (Sigma-Aldrich, India). The 16S rRNA region of bacteria was amplified by Polymerase chain reaction (PCR) using following program: initial denaturing at 94 °C for 3 min, 35 cycles of denaturation at 94 °C for 1 min, annealing at 50 °C for 50 s, elongation at 72 °C for 1:30 min and final extension cycle at 72 °C for 10 min. The PCR conditions of ITS amplification followed the same program with slight change in initial denaturation for 5 min, 35 cycles of denaturation for 30s, annealing at 52 °C.

### 2.3 Phylogenetic analysis

The amplified PCR products were purified using QIAquick® PCR purification kit and sequenced by Sanger sequencing method. The obtained sequence of each isolate was identified using BLASTn (Basic Local Alignment Search Tool) algorithm (www.ncbi.nlm.nih.gov/BLAST) in NCBI (National Center for Biotechnology Information) database. The sequences were deposited to NCBI database and their accession numbers were obtained. Sequences of the individual isolates and the reference sequences were aligned by Clustal W programme [25] and the phylogenetic tree was constructed in MEGA 11.0 by maximum likelihood method, bootstrapped with thousand replications [26].

### 2.4 Screening for plant growth promoting functional traits

All the 79 OTUs obtained were evaluated for the following growth promoting activities.

#### 2.4.1 Indole-3-acetic acid (IAA) production

The production of Indole acetic acid (IAA) by bacterial and fungal isolates was determined in NA broth and PDA broth, respectively, supplemented with L-Tryptophan (500 μg/mL) at 24 h as described by Patten and Glick (2002) [27]. Controls consisted of broth media with microbial inoculation but without tryptophan. After 24 h, bacterial cells and fungal mass were removed by centrifugation of 2 ml of broth at 12,298 g for 2 min. 2 mL of Salkowski's reagent was then added to 1 mL of the supernatant and incubated at room temperature for 30 min. Indoles were indicated by the development of a pink colour and the quantity was determined by measuring the absorbance of supernatant mixture (supernatant + Salkowski's reagent) at 530 nm and comparing with an IAA standard graph plotted for absorbance of commercial IAA ((Sigma-Aldrich, US) at different known concentrations (10, 20, 30, 40, 50, 60, 70, 80, 90, 100 μg/ml).

#### 2.4.2 Phosphate solubilization

Microbial isolates were screened for phosphate solubilization activity following the method of Pikovskaya (1948) [28]. Pikovskaya agar medium was used to prepare the media plates for each bacterial and fungal isolate. Microbial cultures were point inoculated at the four corners of Pikovskaya agar plates and were incubated in incubator at 28 °C for 7 days. The plates were then examined for halo zone formation around each microbial culture and the solubilization index (S.I.) was calculated using the following formula (I) of Moawad and Vleck (1996) [29].

(I) $$S.I=\frac{\text{Colony}\;\text{diameter}+\text{halo}\;\text{zone}\;\text{diameter}}{\text{Colony}\;\text{diameter}}$$

Phosphate solubilization was quantitatively done by growing pure positive isolates in 10 ml of Pikovskaya broth (HiMedia India) at 28 °C for 7 days on a shanking incubator (3 g). After 7 days, 1.5 ml of the broth was centrifuged for 10 min at 12,298 g to obtain the supernatant. 0.1 ml of the supernatant was then reacted with 0.26 ml of Barton's reagent (2.5% ammonium molybedate, 0.126% ammonium metavanadate and 260 ml concentrated nitric acid) [30]. The colour change of supernatant to yellow indicated a positive reaction. Furthermore, the absorbance of the supernatant mixture (supernatant + Barton's reagent) was measured at 430 nm using a UV-VIS spectrophotometer. Phosphate concentration was then measured against a standard curve which was plotted between absorbance and different concentrations of potassium dihydrogen phosphate (KH_2_PO_4_) (10, 20, 30, 40, 50, 60, 90, 100 μg/ml).

#### 2.4.3 Ammonia production

The test for ammonia production was carried out following the protocol of Singh et al. (2014) [31]. Briefly, each microbial isolate was grown in 5 ml of peptone water for 72 h at 30 °C on a shanking incubator (3 g). Microbial cells were removed by centrifugation at 12,298 g for 5 min and Nessler's reagent (K_2_HgI_4_; 1.4%) was added in 2:1 ratio to each culture supernatant. The development of faint to deep yellow to brownish colour indicated the production of ammonia and it was quantified by measuring the absorbance of mixture (peptone broth culture + Nessler's reagent) at 450 nm. Standard curve between different concentrations of ammonium chloride (0.5, 1, 1.5, 2, 2.5, 3, 3.5, 4 mM/ml) and absorbance was used to compute the concentration of ammonia produced by the isolates.

#### 2.4.4 Biocontrol activity

Biocontrol activity was evaluated against two phytopathogens, *Fusarium oxysporum* (a common soil pathogen) and *Botrytis cinerea* (natural enemy of *A. cotula*) [32]. For the assessment of biocontrol activity, dual culture technique was adopted against two pathogens – *B. cinerea* (isolated from grapes) and *F. oxysporum* (isolated from soil) that were identified molecularly. For bacteria, the isolates at log phase were line streaked on one side of PDA plate (2 cm away from periphery) and pathogen was placed equidistant from other side of the plate. For fungi, 5-day old mycelium was inoculated on one side of Petri plate and pathogen was placed equidistant from other side of the plate. The plates were incubated for 4–7 days at 28 °C and percentage inhibition index was calculated by formula (II) as.

(II) $$\%\;\text{Inhibition}=\frac{R1‐R2}{R1}\times 100$$

where, R1 is colony diameter of pathogen in absence of testing isolate (control), and.

R2 is colony diameter of pathogen in presence of testing isolate.

### 2.5 Microcosm experimental setup

Isolated bacterial and fungal strains showing biocontrol activity against the plant pathogen (*B. cinerea*) were cultured in liquid Luria-Bertani (LB) broth for 24 h and PDA broth for 5 days at 28 °C on a rotary shaker (2 g), respectively. The cultures were centrifuged at 12,298 g for 15 min and the pellets were washed twice with sterile water by centrifugation at 12,298 g for 15 min. The bacterial cultures were diluted with sterile water and the suspension was maintained at 10^6^-10^8^ cells ml^−1^ (OD_600nm_ = 0.5) using a spectrophotometer. For fungal inoculum, mycelia pellet was flooded with sterile water and shaken gently to release spores. Equal quantities of strains were added as fungal inoculum, maintained at a concentration of 1 × 10^7^ spores per ml (OD_600nm_ = 0.1) and was amended with 0.2% tween twenty. The pathogen inoculum was prepared separately following the same protocol.

Seeds sterilized using 2% NaOCl for 10 min followed by treatment with streptomycin (100 μg/ml) and cycloheximide (100 μg/ml) for 6 hours [33], were placed on wet sterilized filter papers in sterilized Petri plates. The seeds were incubated at room temperature for 4 days for seed germination. The equal sized seedlings were chosen and inoculated with bacterial and fungal suspensions for 2 h each [34], whereas control was treated with broth without microbial inoculum. Two treated and control seedlings were then planted in pots (2 replicates) filled with triple autoclaved soil and sand in the ratio of 3:1. For efficient inoculation, again 10 ml of microbial inoculum was added to seedlings after 1 week [35]. The whole experiment was carried out in a growth chamber set with 80% relative humidity, 16:8 light: dark photoperiod, 27 °C temperature and irrigated by adding double distilled water.

For inoculation of fungal pathogen, a foliar spray method as used by Posada et al. (2007) was followed [36]. Hand sprayer was used for inoculation of fungal suspension. After 3 weeks, approximately 5 ml of fungal suspension per plant, per inoculation time (twice a week) was sprayed and the relative humidity was maintained at 100% for 24 h. The leaves of the plants were inspected for signs of infection on a daily basis. Whole experiment was repeated thrice.

### 2.6 Data analysis

Relative abundance of each taxon was calculated as the percentage of an isolate belonging to specific taxonomic category compared to the total number of isolates of all categories. Pheatmaps package was used to generate heatmaps for relative abundance of phyla and genera, while as circlize package was used for computing relative abundance of species across sites. Plant growth promoting functional traits were visualized as bar plots using the ggplot2 package. Alpha diversity was measured using Hill number-based species richness (q = 0), Shannon (q = 1, the exponential of Shannon entropy) and Simpson (q = 2, the inverse of Simpson concentration) given their suitability for use in samples obtained by high-throughput sequencing [37]. Besides, rarefaction and extrapolated curves were generated using the “iNEXT” package [38]. Sample-and coverage-based rarefaction and extrapolation curves were generated to examine how diversity increases with increasing sampling effort and completeness. Sample-based curves evaluated the number of individuals in a sample by plotting diversity estimates in relation to the number of sampling units. Coverage-based curves were plotted against rarefied sample completeness to illustrate diversity estimates in relation to sample coverage. All extrapolation curves were plotted using a doubling in sample size, and 500 bootstrap replicates were used to estimate 95% confidence intervals. Ninety-five per cent confidence intervals, a known alternative to standard statistical testing [39], were used to determine if differences were statistically significant. Nonoverlapping 95% confidence intervals, whether rarefied or extrapolated curves are considered to indicate significant differences at a level <5% [40,41].

One-way ANOVA of abundance data across sites and Bray Curtis dissimilarity index for all sites was performed in past software 4.03. The total number of OTUs per site and the number of unique to sites and shared OTUs between the sites is presented in the form of an UpSet plot using Complex Heatmap package. The analyses were carried out using the open-source statistical programming language R 4.1.2 (R Core Team 2022).

## 3 Results

### 3.1 Endophyte species composition

A total of 902 culturable endophytic isolates were obtained from the leaves of *A. cotula*, out of which 800 were bacterial isolates and 102 were fungal isolates. 16s and ITS rDNA sequencing and BLASTn analysis, resolved them into 59 bacterial OTUs and 20 fungal OTUs. The 16s rRNA sequences of bacterial OTUs and ITS sequences of fungal OTUs mostly showed more than 99% sequence similarity with the reference strains (Supplementary Table 1 along with accession numbers). Phylogenetic analysis revealed that these 79 OTUs belonged to 4 bacterial and 2 fungal phyla and 27 bacterial and 14 fungal genera (Fig. 2a, Fig. 2ba and b). Firmicutes was the predominant phyla (29.1%), followed by Proteobacteria (24.1%), Ascomycota (22.8%), Actinobacteria (19%), Zygomycota and Bacteriodetes (2.5% each) (Fig. 3a).

**Fig. 2a:**
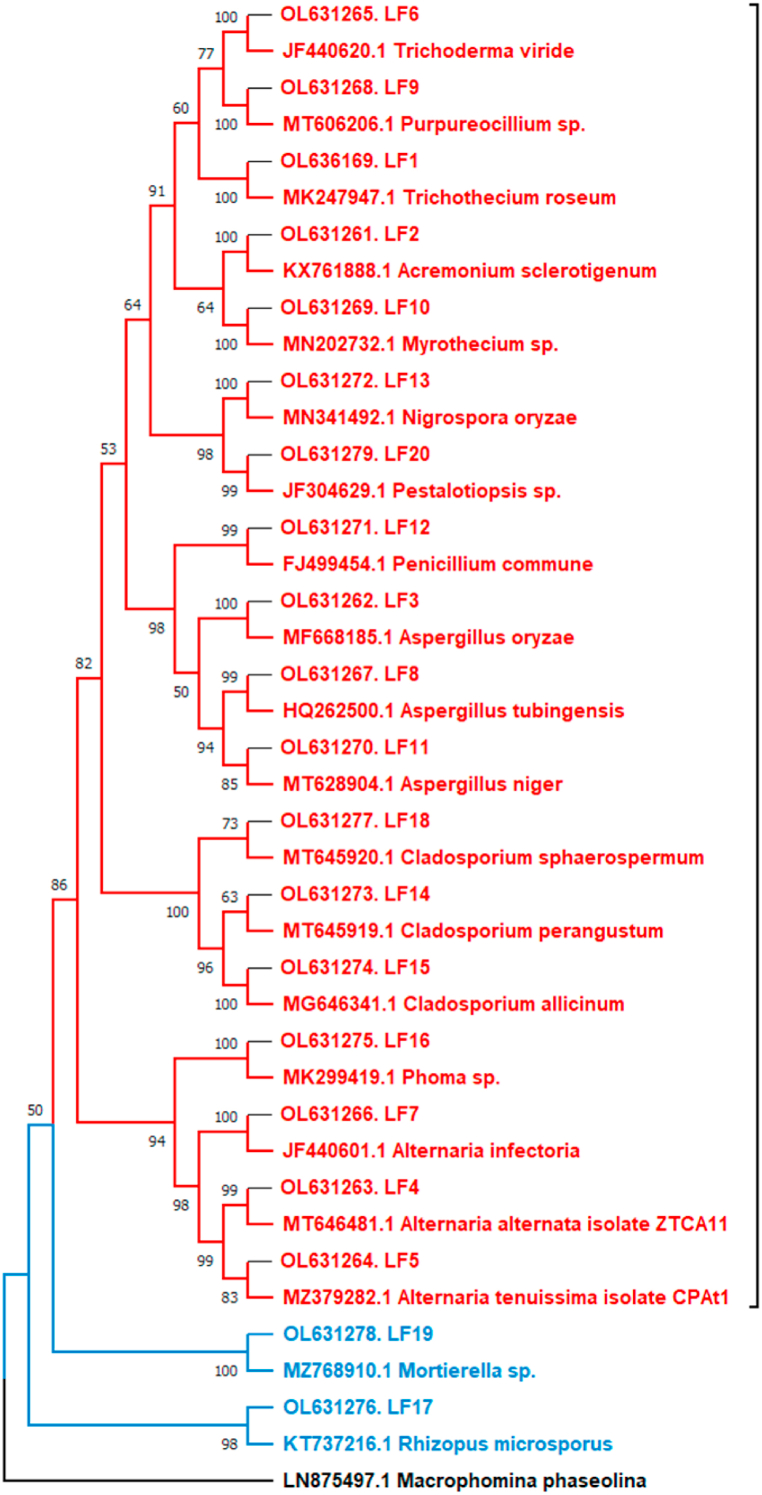
Phylogenetic tree showing the relationship of endophyte ITS gene sequences to representative type species sequences.

**Fig. 2b:**
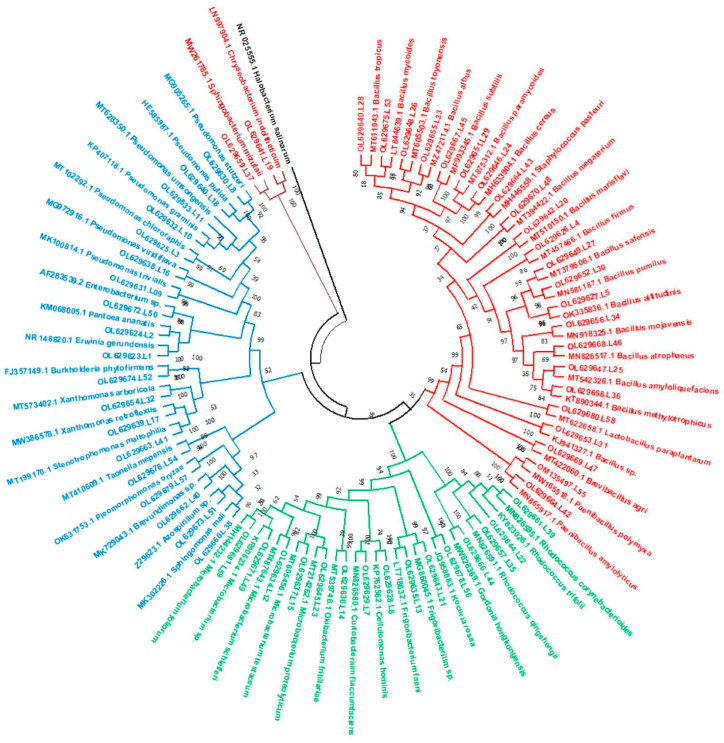
Phylogenetic tree of isolated bacterial endophytes. Red, green, blue and maroon colour represents Firmicutes, Actinobacteria, proteobacteria and Bacteriodetes, respectively. (For interpretation of the references to colour in this figure legend, the reader is referred to the Web version of this article.)

**Fig. 3:**
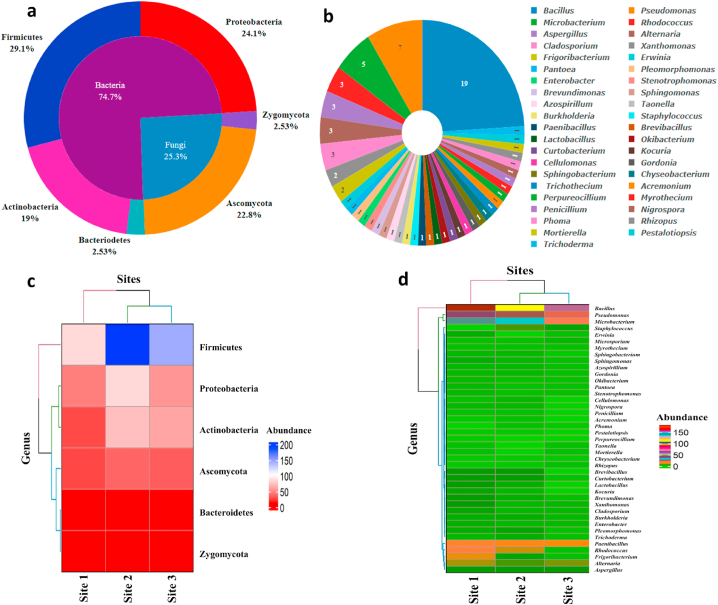
**a**-Pie chart showing relative abundance (%age) of each phylum: **b**- Relative abundance %age of each genus **c**- Heat map showing the relative abundance of each phylum across sites: **d**- Heat map showing relative abundance of genera across sites.

In the dominant phyla of Firmicutes (23 OTUs), bacteria belonged to single class bacilli, mainly consisted of genus *Bacillus* (18 out of 23 OTUs-78.26%). Proteobacteria (19 OTUs), the second most abundant phylum included bacteria belonging to class Gamma-proteobacteria, Alpha-proteobacteria and Beta-proteobacteria and greater portion of them were obtained from Gamma-Proteobacteria (15 out of 19 OTUs- 78.94%). OTUs from phyla Actinobacteria (15 OTUs) included representative members of genera *Microbacterium* (33.33% of 15), *Rhodococcus* (20% of 15), *Frigoribacterium* (13.33% of 15), *Okibacterium, Kokuria, Cellulomonas* and *Gordonia* (6.66% of 15 each). Ascomycota (18 OTUs) comprised fungi belonging to class Sordariomycetes (7 of 18 OTUs-38.88%), Dothideomycetes (7 of 18 OTUs-38.88%) and Eurotiomycetes (4 out of 18 OTUs-22.22%). Phyla Zygomycota and Bacteriodetes were represented by only two OTUs in each (Supplementary Table 2).

At the generic level, *Bacillus* was the dominant genus (18 of 79 OTUs- 24.1%), followed by *Pseudomonas* (7 of 79–8.9%) and *Microbacterium* (5 of 79–6.3%). Abundance of all other genera ranged between 3.8 and 1.3% (Fig. 3b). Least abundant genera were *Lactobacillus, Brevibacillus, Erwinia, Pantoea, Pleomorphomonas, Enterobacter, Stenotrophomonas*, *Brevundimonas, Spingomonas, Sphingobacterium, chryseobacterium, Azospirillum*, *Taonella, Burkholderia*, *Okibacterium, Kokuria, Cellulomonas*, *Gordonia, Trichothecium, Acremonium, Perpureocillium, Myrothecium, Nigrospora, Mortierella, Rhizopus, Pestalotiopsis, Pencillium* and *Trichoderma.* At species level, *Bacillus cereus* and *Bacillus mojavensis* were most abundant (28 isolates each- 3.1% of total isolates), followed by *Bacillus firmus* and *Bacillus safensis* (26 isolates each – 2.8% of total isolates). Abundance of other species ranged between 2.7 and 0.2%. The least abundant species were *Cladosporium allicinum* and *Perpureocillium lilacinum* represented by single isolate.

### 3.2 Species diversity

Richness based on genera and species varied across sites in respect of both fungal and bacterial communities, although no such obvious difference in richness was noticed at higher taxonomic levels. 4 phyla, 6 classes, 14 orders, 17 families of bacteria and 2 phyla, 4 classes, 8 orders, 10 families of fungi were recorded in all the sites. Phylum Firmicutes was abundant at all the sites but was relatively more abundant at site 1, followed by site 2 and site 3. The phylum Proteobacteria was most abundant at sites 1 and 3 while as Actinobacteria was dominant at site 2 (Fig. 3c).

The richness of genera also differed between the sites with highest number recorded at site 1 (26 bacterial genera and 13 fungal genera of total 41genera), followed by site 2 (26 bacterial genera and 12 fungal genera of total 41 genera) and site 3 (22 bacterial genera and 9 fungal genera of total 41genera) (Fig. 3d).

**Fig. 4:**
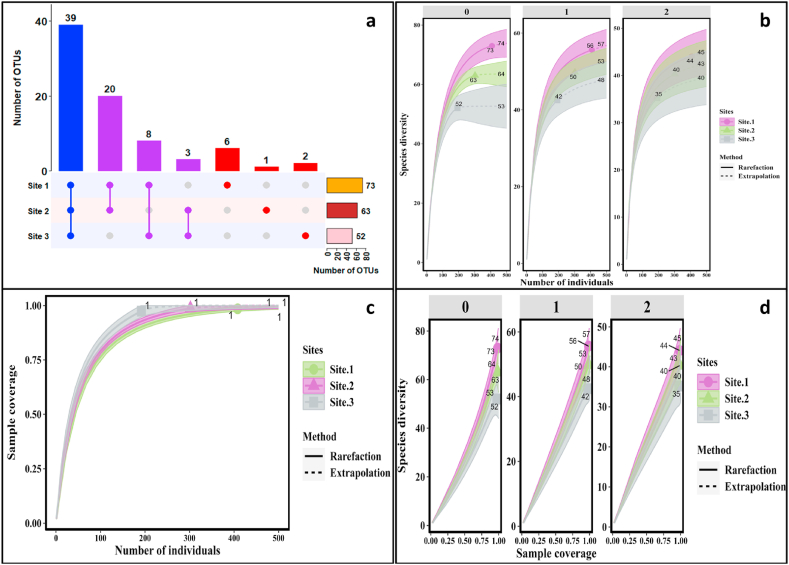
**a-**Upset diagram showing the total, shared and specific OTUs among three sites, **b-** Sample-size-based rarefaction and extrapolation of the diversity measure for order q = 0, q = 1, and q = 2, **c-** Sample-size-based rarefaction (solid lines) and extrapolation (dotted lines) of OTUs from three sites, **d-** Coverage-based rarefaction (solid lines) and extrapolation (dotted lines) for the diversity measure for order q = 0–2.

In all the study sites, *Bacillus* was the most abundant genus across all sites. However, it was more abundant at site 1 followed by site 2 and site 3, respectively. However, within genus *Bacillus*, *B. toyonensis* (24 isolates) was abundant at site 1, *B. pumilus* and *B. velezensis* at site 2 (14 isolates each) and *B. atropheus* at site 3 (14 isolates). Furthermore, among the total identified OTUs, 39 OTUs were shared between all the sites; 20, 8 and 3 OTUs were shared between sites 1 and 2, 1 and 3, and 2 and 3, respectively. Only 5 OTUs were specific to site 1, 2 OTUs to site 3 and only 1 OTU was specific to site 2 (Fig. 4a). This OTU overlap pattern indicated a close relationship between endophytic communities and the host from three different study sites.

**Fig. 5:**
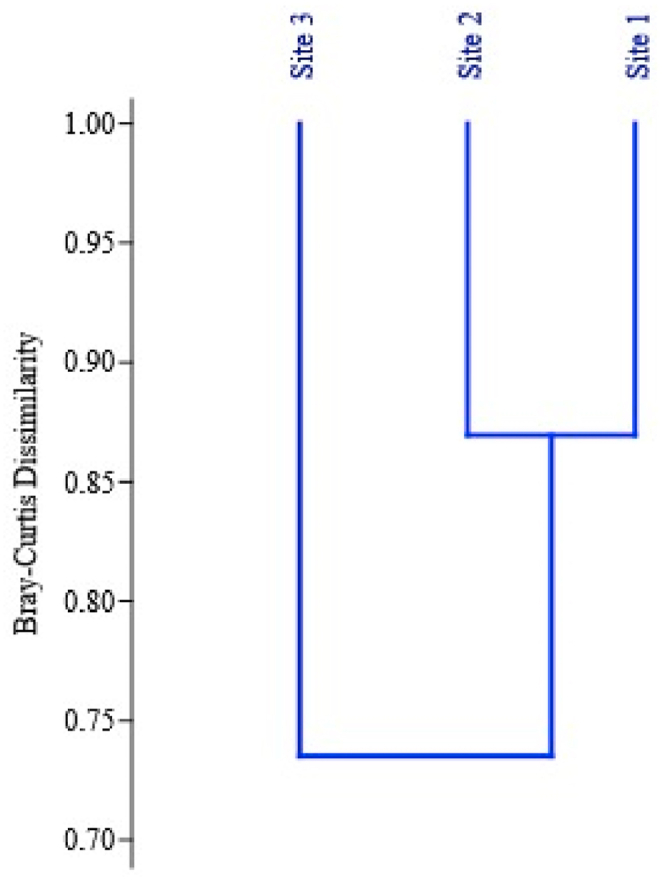
Clustering of sites based on Bray-Curtis cluster analysis. Sites in the same clade represent similar species composition.

All the diversity curves (Species richness (q = 0), Shannon (q = 1) and Simpson (q = 2)) illustrate that the microbial endophytes exhibited their highest diversity in site 1 followed by site 2 and then site 3 (Fig. 4b, c, 4d). The result presented in show significant differences in species richness across sites but differences in Shannon and Simpson's indices were not statistically significant. To further unravel the degree of similarity between sites based on species composition, Bray-Curtis cluster analysis was performed which resulted in two groups separated at a vertical distance of 0.88 and one group comprised site 1 and 2 and the other group included only site 3 (Fig. 5). The results revealed that site 1 and site 2 had more similarity in species composition as compared to site 3.

### 3.3 Plant growth promoting traits

#### 3.3.1 IAA production

**Fig. 6:**
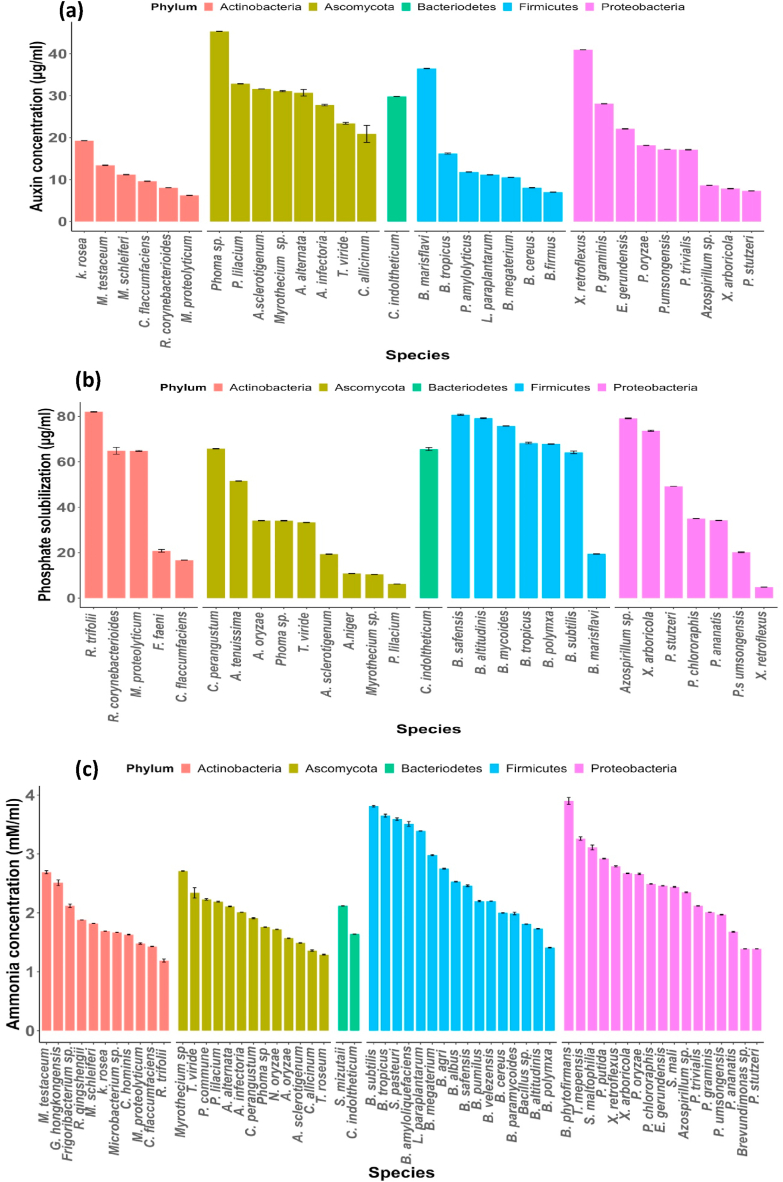
Bar plots show the activity range of different endophytes, with different colours indicating the phyla to which each species belongs. Bars show standard errors of the means (SE, for n = 3). Figures **a**, **b,** and **c** summarize the graphs depicting the IAA production, phosphate solubilization, and ammonia production, respectively. (For interpretation of the references to colour in this figure legend, the reader is referred to the Web version of this article.)

Out of the 79 OTUs assayed, 39.24% OTUs showed ability to synthesize IAA using L-tryptophan as a precursor and produced IAA in the range of 45.33–6.24 μg/ml. OTUs belonging to phyla Ascomycota showed highest IAA production which ranged from 45.33 to 20.90 μg/ml, followed by Proteobacteria (40.94–7.31 μg/ml), Firmicutes (36.50–7.01 μg/ml), Actinobacteria (19.26–6.24 μg/ml) and Bacteriodetes (29.82 μg/ml) (Fig. 6a). Among all the IAA positive isolates, *Phoma* sp. showed the highest activity (45.33 μg/ml), followed by *Xanthomonas retroflexus* (40.33 μg/ml) while as least activity was shown by *Microbacterium proteolyticum* (6.24 μg/ml).

#### 3.3.2 Phosphate solubilization

The ability of different OTUs to solubilize the inorganic phosphate was first confirmed on Pikovskaya agar plate. Only 29 OTUs showed positive results with solubilization index (SI) of 2.75mm–1.06 mm. Quantity of phosphate solubilization ranged from 80.70 to 4.84 μg/ml. Highest activity was shown by *R. trifolii* (82 μg/ml), followed by *B. safensis* (80.70 μg/ml), *B. altitudinis* (79.20 μg/ml), *Azospirillum* sp. (79.11 μg/ml) while as least activity was shown by *Xanthomonas retroflexus* (4.84 μg/ml). Most of the phosphate solubilizing OTUs belonged to division Ascomycota (9 OTUs), followed by Firmicutes and Proteobacteria (7 OTUs each), Actinobacteria (5 OTUs) and Bacteriodetes (1 OTU) (Fig. 6b).

#### 3.3.3 Ammonia production

Most of the OTUs evaluated (59 out of 79) showed ammonia production activity with values ranging from 3.90 to 1.19 mM/ml. Isolates showing the positive activity mostly belonged to phylum Proteobacteria (17 OTUs) and produced ammonia in the range of 3.90–1.39 μg/ml, followed by Firmicutes (16 OTUs- 3.81-1.41 mM/ml), Ascomycota (13 OTUs- 2.71-1.36 mM/ml), Actinobacteria (11 OTUs- 2.69-1.43 mM/ml) and Bacteriodetes (2 OTUs- 2.12 & 1.64 mM/ml). Overall, highest production was recorded with *Bulkholderia phytofirmans* of phylum Proteobacteria (3.90 mM/ml), followed by *Bacillus subtilis* of phyla Firmicutes (3.81 mM/ml) and least was obtained from *Rhodococcus trifolii* of phylum Actinobacteria (1.73 mM/ml) (Fig. 6c).

#### 3.3.4 Biocontrol activity

All 79 OTUs underwent screening for their antagonistic activity against *Fusarium oxysporum* and *Botrytis cinerea*, by assessing percentage of inhibition zone formation in dual culture tests. Only 16 OTUs (20.26%) exhibited activity at least against one of the pathogens. Higher number of OTUs that exhibited biocontrol activity belonged to Phylum Proteobacteria and Ascomycota (6 of 16 OTUs- 37.5% each), followed by Firmicutes (2 OTUs), Actinobacteria and Bacteriodetes (1 OTU each). 14 OTUs were effective against *Fusarium* and 7 against *Botrytis*. Only 6 OTUs were active against both the pathogens. Among the effective OTUs, 9 belonged to bacteria and 7 to fungi. Among the bacteria, 9 were effective against *Fusarium* and 5 against *Botrytis* and only 4 showed positive results against both pathogens including *Pseudomonas stutzeri*, *Azospirillum* sp., *Enterobacter* sp. and *Chryseobacterium indoltheticum*. Among fungi, 6 were effective against *Fusarium* and 2 against *Botrytis* while only two were effective against both the pathogens, including *Trichothecium roseum* and *Trichoderma viride*.

In general, bacteria exhibited greater inhibitory activity against *Botrytis*, with the most significant inhibition of 52.20% was observed with bacterium *Pseudomonas stutzeri*. For *Fusarium*, the fungal OTUs demonstrated more inhibitory activity, with *Trichothecium roseum* recording the highest inhibition of 49.11% against *Fusarium* (Table 2).

**Table 2:** Plant growth promoting activities of isolated endophytes.

Sample ID	Accession No.	Species_Name	Auxin Production (μg/ml)	Phosphate Solubilization (μg/ml)	Ammonia Production (mM/ml)	Biocontrol against *Botrytis* (%age Inhibition)	Biocontrol against *Fusarium* (%age Inhibition)
Proteobacteria							
L1	OL629623	*Erwinia gerundensis*	22.11 ± 0.082		2.46 ± 0.004		
L2	OL629624	*Pantoea ananatis*		34.20 ± 0.09	1,68 ± 0.008		40.48 ± 2.06
L3	OL629626	*Pseudomonas chlororaphis*		34.99 ± 0.03	2.49 ± 0.005		
L8	OL629630	*Pseudomonas stutzeri*	7.31 ± 0.023	49.22 ± 0.01	1.39 ± 0.002	52.20 ± 1.09	29.17 ± 2.06
L9	OL629631	*Pseudomonas trivialis*	17.12 ± 0.079		2.12 ± 0.004		27.98 ± 2.73
L10	OL629632	*Pseudomonas graminis*	28.11 ± 0.029		2.01 ± 0.002		
L11	OL629633	*Pseudomonas umsongensis*	17.20 ± 0.021	20.17 ± 0.16	1.97 ± 0.006		
L16	OL629638	*Pseudomonas viridiflava*					
L17	OL629639	*Xanthomonas retroflexus*	40.94 ± 0.031	4.84 ± 0.08	2.79 ± 0.011		
L18	OL629640	*Pseudomonas putida*			2.92 ± 0.006	28.49 ± 0.680	
L32	OL629654	*Xanthomonas arboricola*	7.85 ± 0.071	73.65 ± 0.23	2.67 ± 0.005		
L38	OL629660	*Sphingomonas mali*			2.44 ± 0.007		
L40	OL629662	*Brevundimonas* sp.			1.39 ± 0.003		
L41	OL629663	*Stenotrophomonas maltophilia*			3.11 ± 0.041		
L51	OL629673	*Azospirillum* sp.	8.65 ± 0.022	79.11 ± 0.16	2.35 ± 0.009	41.82 ± 0.45	36.90 ± 2.06
L50	OL629672	*Enterobacter* sp.				48.43 ± 1.09	36.61 ± 1.55
L54	OL629676	*Taonella mepensis*			3.26 ± 0.029		
L52	OL629674	*Burkholderia phytofirmans*			3.90 ± 0.058		
L56	OL629677	*Pleomorphomonas oryzae*	18.17 ± 0.042		2.66 ± 0.012		
Firmicutes							
L4	OL629626	*Bacillus firmus*	7.01 ± 0.038				
L5	OL629627	*Bacillus altitudinis*		79.20 ± 0.17	1.73 ± 0.003		
L20	OL629642	*Bacillus marisflavi*	36.50 ± 0.040	19.44 ± 0.12			
L24	OL629646	*Bacillus cereus*	8.06 ± 0.067		2.00 ± 0.003		
L25	OL629647	*Bacillus amyloliquefaciens*			3.51 ± 0.040		
L26	OL629648	*Bacillus toyonensis*					
L27	OL629649	*Bacillus safensis*		80.70 ± 0.27	2.46 ± 0.014		
L28	OL629650	*Bacillus tropicus*	16.21 ± 0.135	68.24 ± 0.34	3.65 ± 0.026		
L29	OL629651	*Bacillus paramycoides*			1.99 ± 0.021		
L30	OL629652	*Bacillus pumilus*			2.20 ± 0.010		28.57 ± 1.79
L31	OL629653	*Bacillus* sp.			1.81 ± 0.003		
L33	OL629655	*Bacillus albus*			2.53 ± 0.005		
L34	OL629656	*Bacillus mojavensis*					
L42	OL629664	*Paenibacillus amylolyticus*	11.82 ± 0.027				31.26 ± 2.36
L45	OL629667	*Bacillus subtilis*		64.14 ± 0.58	3.81 ± 0.013		
L46	OL629668	*Bacillus atrophaeus*					
L47	OL629669	*Brevibacillus agri*			2.75 ± 0.007		
L36	OL629658	*Bacillus velezensis*			2.20 ± 0.002		
L48	OL629670	*Bacillus megaterium*	10.57 ± 0.015		2.98 ± 0.010		
L53	OL629675	*Bacillus mycoides*		75.76 ± 0.07			
L55	OL629678	*Bacillus polymyxa*		67.84 ± 0.12	1.41 ± 0.005		
L43	OL629665	*Staphylococcus pasteuri*			3.59 ± 0.021		
L57	OL629679	*Lactobacillus paraplantarum*	11.17 ± 0.045		3.39 ± 0.003		
Actinobacteria							
L58	OL629680	*Microbacterium foliorum*					
L59	OL629681	*kocuria rosea*	19.26 ± 0.048		1.69 ± 0.003		
L49	OL629671	*Microbacterium* sp.			1.67 ± 0.002		
L44	OL629666	*Gordonia hongkongensis*			2.51 ± 0.049		
L39	OL629661	*Rhodococcus corynebacterioides*	8.07 ± 0.021	64.84 ± 1.53			
L35	OL629657	*Rhodococcus qingshengii*			1.88 ± 0.001		28.57 ± 1.79
L21	OL629643	*Frigoribacterium* sp.			2.12 ± 0.030		
L22	OL629644	*Rhodococcus trifolii*		82.00 ± 0.17	1.19 ± 0.726		
L23	OL629645	*Microbacterium proteolyticum*	6.24 ± 0.044	64.75 ± 0.22	1.48 ± 0.013		
L12	OL629634	*Microbacterium schleiferi*	11.20 ± 0.049		1.82 ± 0.002		
L13	OL629635	*Frigoribacterium faeni*		20.78 ± 0.63			
L14	OL629636	*Okibacterium fritillariae*					
L15	OL629637	*Microbacterium testaceum*	13.42 ± 0.060		2.69 ± 0.027		
L6	OL629628	*Cellulomonas hominis*			1.63 ± 0.006		
L7	OL629629	*Curtobacterium flaccumfaciens*	9.63 ± 0.048	16.67 ± 0.02	1.43 ± 0.004		
Bacteriodetes							
L19	OL629659	*Sphingobacterium mizutaii*			2.12 ± 0.003		
L37	OL629641	*Chryseobacterium indoltheticum*	29.82 ± 0.017	65.60 ± 0.67	1.64 ± 0.002	23.27 ± 1.09	32.82 ± 2.13
Ascomycota							
LF1	OL636169	*Trichothecium roseum*			1.29 ± 0.007	26.56 ± 0.96	49.11 ± 2.36
LF2	OL631261	*Acremonium sclerotigenum*	31.62 ± 0.03	19.33 ± 0.13	1.49 ± 0.006		
LF3	OL631262	*Aspergillus oryzae*		34.10 ± 0.10	1.57 ± 0.004		
LF4	OL631263	*Alternaria alternate*	30.71 ± 0.78		2.11 ± 0.005		44.64 ± 1.79
LF5	OL631264	*Alternaria tenuissima*		51.58 ± 0.11			
LF6	OL631265	*Trichoderma viride*	23.42 ± 0.21	33.34 ± 0.11	2.34 ± 0.088	48.33 ± 1.67	46.43 ± 1.79
LF7	OL631266	*Alternaria infectoria*	27.77 ± 0.19		2.01 ± 0.392		
LF8	OL631267	*Aspergillus tubingensis*					
LF9	OL631268	*Perpureocillium lilacium*	32.86 ± 0.05	6.22 ± 0.01	2.19 ± 0.009		47.62 ± 2.06
LF10	OL631269	*Myrothecium* sp.	31.11 ± 0.14	10.43 ± 0.06	2.71 ± 0.003		36.31 ± 2.73
LF11	OL631270	*Aspergillus niger*		10.81 ± 0.06			
LF12	OL631271	*Penicillium commune*			2.23 ± 0.383		
LF13	OL631272	*Nigrospora oryzae*			1.72 ± 0.003		
LF14	OL631273	*Cladosporium perangustum*		65.79 ± 0.11	1.91 ± 0.009		45.83 ± 2.73
LF15	OL631274	*Cladosporium allicinum*	20.90 ± 0.02		1.36 ± 0.011		
LF16	OL631275	*Phoma* sp.	45.33 ± 2.04	34.07 ± 0.15	1.76 ± 0.014		

Among the evaluated endophytes, 68 showed one or other activity. Only 5 endophytes showed all the 4 activities, 12 were positive for 3 activities, 26 showed at least 2 activities while 26 showed only 1 activity (Supplementary Fig. S6). Overall, most of the endophytes with positive result for at least one of the activity belonged to phyla Firmicutes (20 out of 68), followed by Proteobacteria (18 out of 68) and Ascomycota (15 out of 68) (Table 2).

### 3.4 Microcosm assay

The results indicate that plants that were not inoculated with the antagonistic isolates (*Pseudomonas stutzeri*, *Pseudomonas putida*, *Azospirillum* sp., *Enterobacter* sp., *Chryseobacterium indoltheticum*, *Trichothecium roseum, Trichoderma viride*) exhibited premature leaf abscission starting as early as the third day after being sprayed with the pathogen *Botrytis cinerea*. These plants eventually succumbed to *B. cinerea* infection within 12 days. On the other hand, plants that were inoculated with the antagonistic isolates showed normal growth in terms of vegetative parameters and displayed little or no disease symptoms after being sprayed with the pathogen. This suggests that the antagonistic isolates provided significant protection to the plants against *B. cinerea* infection.

## 4 Discussion

Leaves of a plant represent the most peculiar habitat that is frequently exposed to numerous environmental impulses, including temperature fluctuations, relative humidity, desiccation, ultraviolet radiations and apoplastic nutrient stress [42]. In our study, we were successfully able to isolate 79 OTUs, of which 59 were bacterial and 20 were fungal OTUs. The number of isolates obtained was relatively higher compared to previous studies [[43], [44], [45]] which could be attributed to the utilization of two isolation techniques (fragmentation and serial dilution). The most abundant bacterial phylum identified was Firmicutes (represented by class Bacilli), accounting for 29.1% of the bacterial isolates. Proteobacteria was the second most abundant phylum, primarily represented by the class Gamma-proteobacteria. This finding is consistent with some previous studies [[46], [47], [48]] but differs from others that reported Proteobacteria as the dominant phylum in leaf endophytic bacteria [49,50]. Among the fungal isolates, the majority belonged to the phylum Ascomycota (90%), with classes Sordariomycetes (35%) and Dothideomycetes (30%) being the most represented. This observation aligns with other similar studies [[51], [52], [53]]. Several bacterial genera (*Stenotrophomonas, Taonella, Pleomorphomonas, Bravibacillus, Lactobacillus, Gordonia, Frigoribacterium, Okibacterium,* and *Cellulomonas*) were reported for the first time as leaf endophytes of an invasive plant species. However, they have been previously documented in leaves of other plants [54,55]. Among the fungi, 11 out of 14 genera have been previously documented [56], while *Trichothecium*, *Acremonium*, and *Nigrospora* were reported for the first time as foliar endophytic fungi in an invasive plant species. The majority of OTUs, both from fungi and bacteria, were associated with the genus *Bacillus*, accounting for 22.78% of the total isolates. This finding is consistent with a similar observation in the invasive plant species *Senecio vulgaris* [57].

In our study, we observed differences in the composition of leaf endophytic communities among the different sites. Sites 1 and 2 showed relatively similar endophytic diversity, while site 3 exhibited distinct differences (Fig. 5). This variation in microbial communities may be attributed to the fact that leaves represent an open niche directly exposed to environmental fluctuations, which can influence the composition of the microbial community. It has been previously observed that there is a negative correlation between the similarity of leaf-inhabiting microflora and geographical distance [58,59]. This dissimilarity can be attributed to the fact that the acquisition of microbiome by plants in natural ecosystems is primarily influenced by local conditions, such as soil, animals, wind, and rainfall [42]. It is well-documented that the similarity of climatic factors tends to decrease as the geographical distance between sites increases, resulting in an increased dissimilarity in microbial assemblages between distant sites [[57], [58], [59]]. This distance-decay relationship could explain the similarity in endophytic communities between sites 1 and 2, which are closer to each other compared to site 3. One interesting finding in our study is the dominance of the genus *Bacillus* across all study sites. *Bacillus* is known for its ability to produce endospores and its strong environmental adaptability. These characteristics may contribute to the widespread presence and abundance of *Bacillus* in the leaf endophytic communities investigated [60,61].

We also discovered that the majority of the OTUs obtained from *A. cotula* leaves exhibited plant growth promoting activities. The most common activity observed was ammonia production, which was present in 74.68% of the isolates, with *Bacillus* and *Pseudomonas* being the dominant genera. Similar findings have been reported in *Camellia sinensis* by Hazarika et al. (2021) [62]. Ammonia production by microbes can provide nitrogen to host plants, promoting their growth and acting as a defense mechanism against plant pathogens [63]. Furthermore, 39.24% of the OTUs were capable of producing indole acetic acid (IAA) in the presence of L-tryptophan. Among them, *Phoma* sp. showed the highest production of IAA, which is consistent with reports in *Glycyrrhiza glabra* [64]. *Bacillus* and *Pseudomonas* were the predominant genera among the IAA-producing isolates, which align with similar findings in *Oryza sativa* [65,66], *Teucrium polium* [63], and *Pulicaria incise* [67]. IAA production has a direct impact on various physiological processes in plants, including photosynthesis, pigment formation, and metabolite biosynthesis [68]. It also plays a crucial role in establishing mutualistic interactions between endophytes and host plants [63]. Additionally, 36.70% of the OTUs exhibited phosphorous solubilization activity, with *R. trifolii*, *B. safensis*, *B. altitudinis*, and *Azospirillum* sp. identified as the highest phosphate solubilizers. These microbes have also been reported as phosphate solubilizers by other researchers [42,69]. Inorganic phosphate solubilizers enhance the availability of phosphorous, which is essential for key plant functions such as nutrient uptake, photosynthesis, and energy transfer [62,70]. Overall, the study highlights the prevalence of plant growth promoting activities among the endophytic microbial communities associated with *A. cotula* leaves. The production of ammonia, IAA, and phosphorous solubilization by these endophytes suggests their potential contribution to the growth and development of the host plant, as well as their role in establishing beneficial interactions.

In this study, we also investigated the inhibitory effect of seven endophytic OTUs against *Botrytis cinerea*, a pathogen known to cause severe damage to *A. cotula* plants during wet autumn in its native region [32]. This pathogen has been observed to limit the growth of *A. cotula.* Through experimental studies, we demonstrated that *A. cotula* plants inoculated with endophytic isolates showing biocontrol activity against *B. cinerea* were able to survive successfully compared to control plants that were not inoculated. This indicates that the presence of these specific endophytic isolates provided protection to the plants against the pathogen.

## 5 Conclusion

The study focuses on the microbial diversity associated with the leaves of *A. cotula*, a plant species found in the Kashmir Himalaya region of Jammu and Kashmir, India. It provides valuable insights into the composition and functioning of cultivable microbial communities, specifically endophytes, inhabiting the leaves of this plant. The comparative analysis of the bacterial 16s rRNA and fungal ITS sequencing data to assess the diversity of endophytic microorganisms at three different sites revealed a high level of diversity among the microbial communities found within the leaves of *A. cotula*. Furthermore, the study identified several microbial isolates with plant growth promoting activities, particularly the biocontrol activity against *B. cinerea*, an enemy of *A. cotula* within its native range. This suggests that these endophytic microbial groups play a role in enhancing the competitiveness and rapid growth of *A. cotula* in Kashmir Himalaya. The findings of this study lay the foundation for further understanding the relationship between the invasive host plant and its microbial symbionts. By deciphering the interactions between the invasive plant and its associated microorganisms, insights may be gained that could enable the development of strategies to disrupt or manipulate specific symbiotic relationships for development of targeted approaches to manage invasive alien plant species.

## Funding

The authors would like to acknowledge the financial support provided by 10.13039/501100001412Council of Scientific and Industrial Research (CSIR), India/10.13039/501100001501University Grants Commission (UGC), India for providing fellowship as JRF/SRF.

## Data availability

The datasets have been deposited in the NCBI gene bank under accession number OL629623 - OL629681, OL631261- OL631279, OM135497 and OL636169.

## CRediT authorship contribution statement

**Iqra Bashir:** Writing – review & editing, Writing – original draft, Methodology, Conceptualization. **Aadil Farooq War:** Writing – review & editing, Writing – original draft, Methodology, Conceptualization. **Iflah Rafiq:** Writing – review & editing. **Zafar A. Reshi:** Writing – review & editing, Supervision, Methodology, Formal analysis, Data curation, Conceptualization. **Irfan Rashid:** Writing – review & editing, Supervision, Methodology, Formal analysis, Data curation, Conceptualization. **Yogesh S. Souche:** Writing – review & editing, Supervision, Methodology.

## Declaration of competing interest

The authors declare that they have no known competing financial interests or personal relationships that could have appeared to influence the work reported in this paper.
