# Supplementary material for: Uncovering the secret weapons of an invasive plant: The endophytic microbes of Anthemis cotula
Source: Heliyon. 2024 Apr 19;10(9):e29778. doi: 10.1016/j.heliyon.2024.e29778 (PMC11058297; doi:10.1016/j.heliyon.2024.e29778)
Supplement: Multimedia component 1 [file mmc1.docx]

**Uncovering the Secret Weapons of an Invasive Plant: The Endophytic Microbes of *Anthemis cotula***

Iqra Bashir^a^*, Aadil Farooq War^a^, Iflah Rafiq^a^, Zafar A. Reshi^a^, Irfan Rashid^a^, Yogesh S. Shouche^b^

^a^Department of Botany, University of Kashmir, Srinagar-190006, Jammu and Kashmir, India.

^b^Azim PremJi University Bengaluru, Karnataka, India.

***Corresponding author:** Iqra Bashir

**E-mail Id:** [Iqramir.scholar@kashmiruniversity.net](mailto:Iqramir.scholar@kashmiruniversity.net)

**Telephone Number:** +91-7889772038

**16-digit ORCID:** 0000-0001-8803-4755


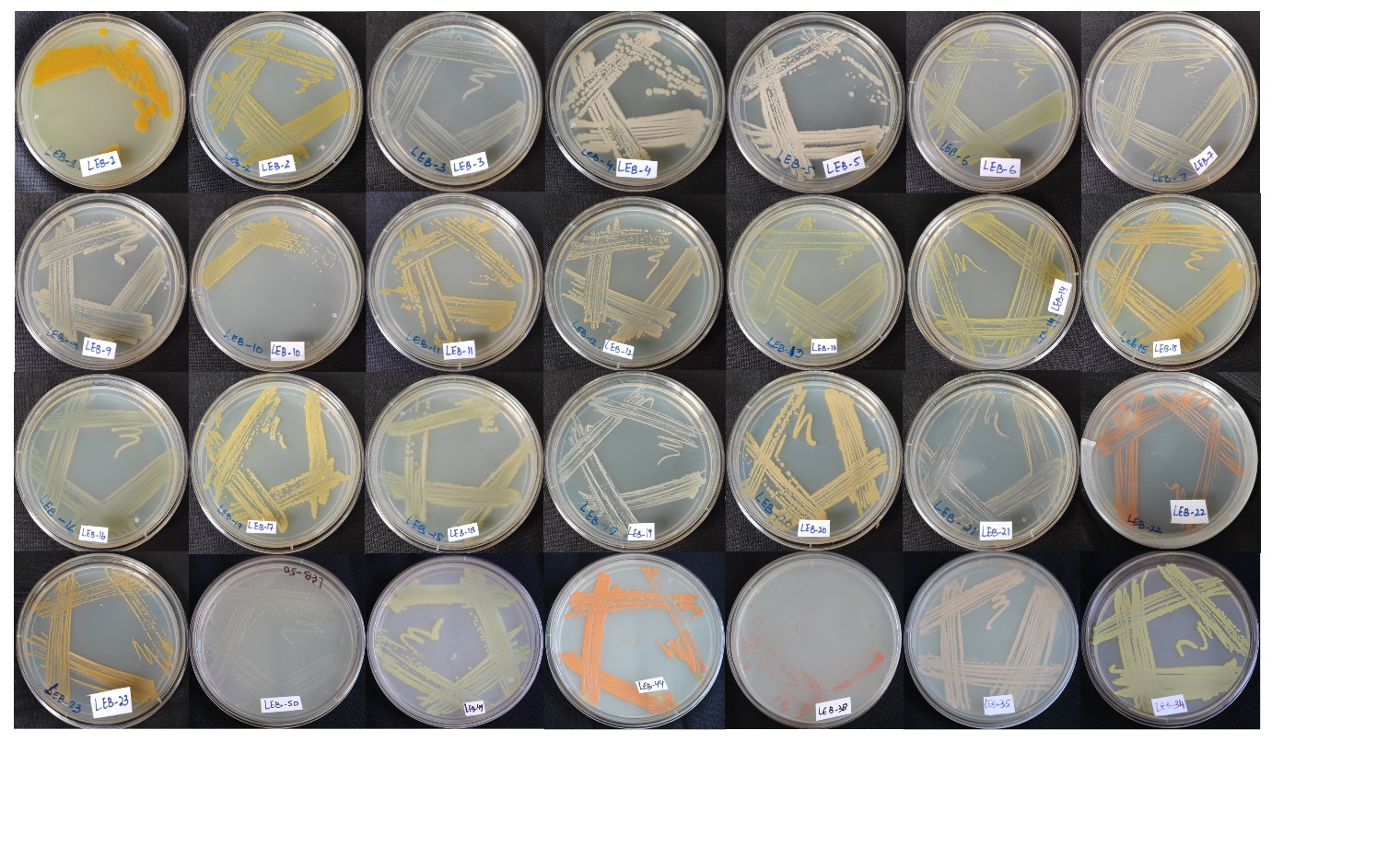


**Supplementary figure S1: Culturable endophytic bacterial endophytes isolated from phyllosphere of *A. cotula***


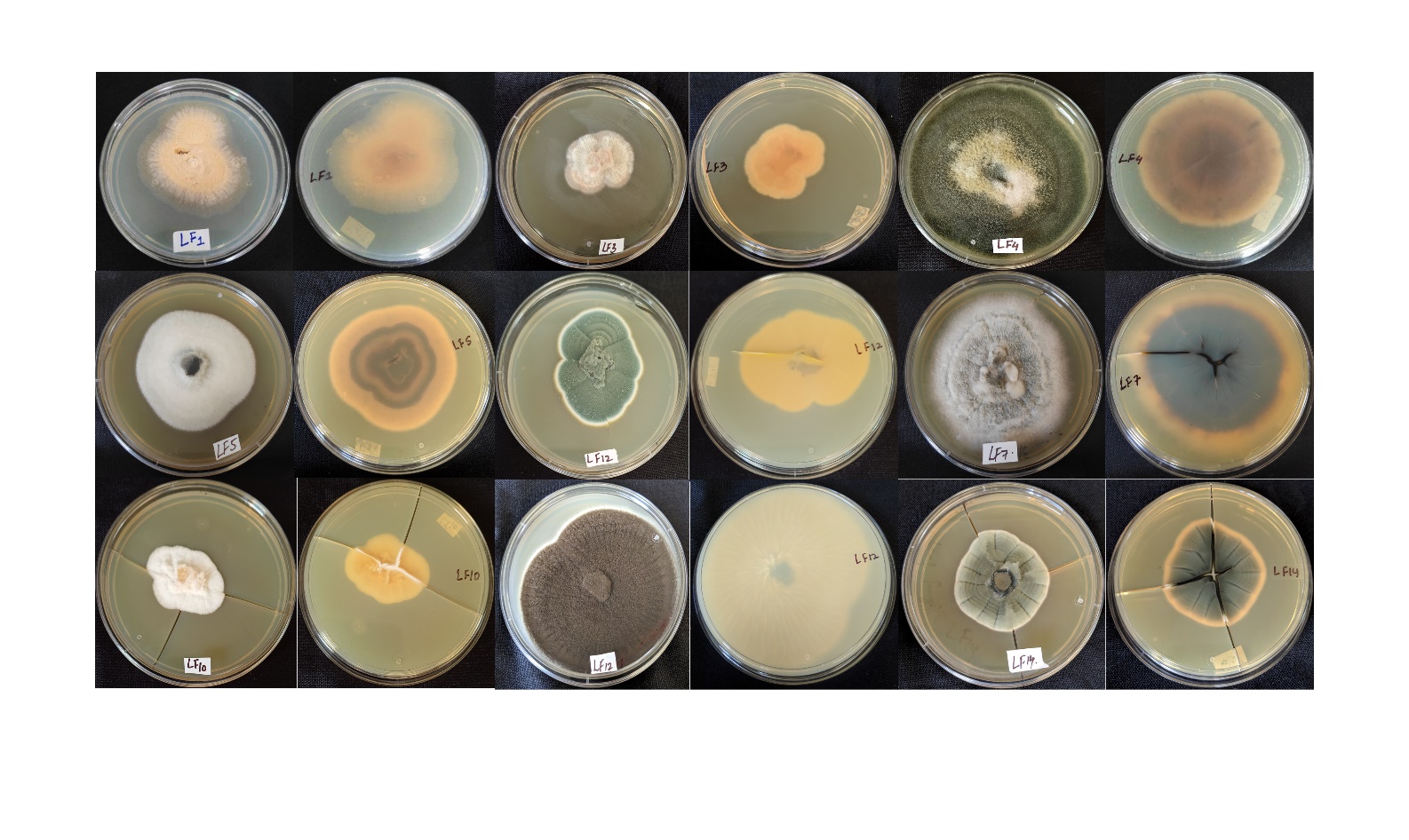


**Supplementary figure S2: Culturable fungal endophytes isolated from phyllosphere of *A. cotula***


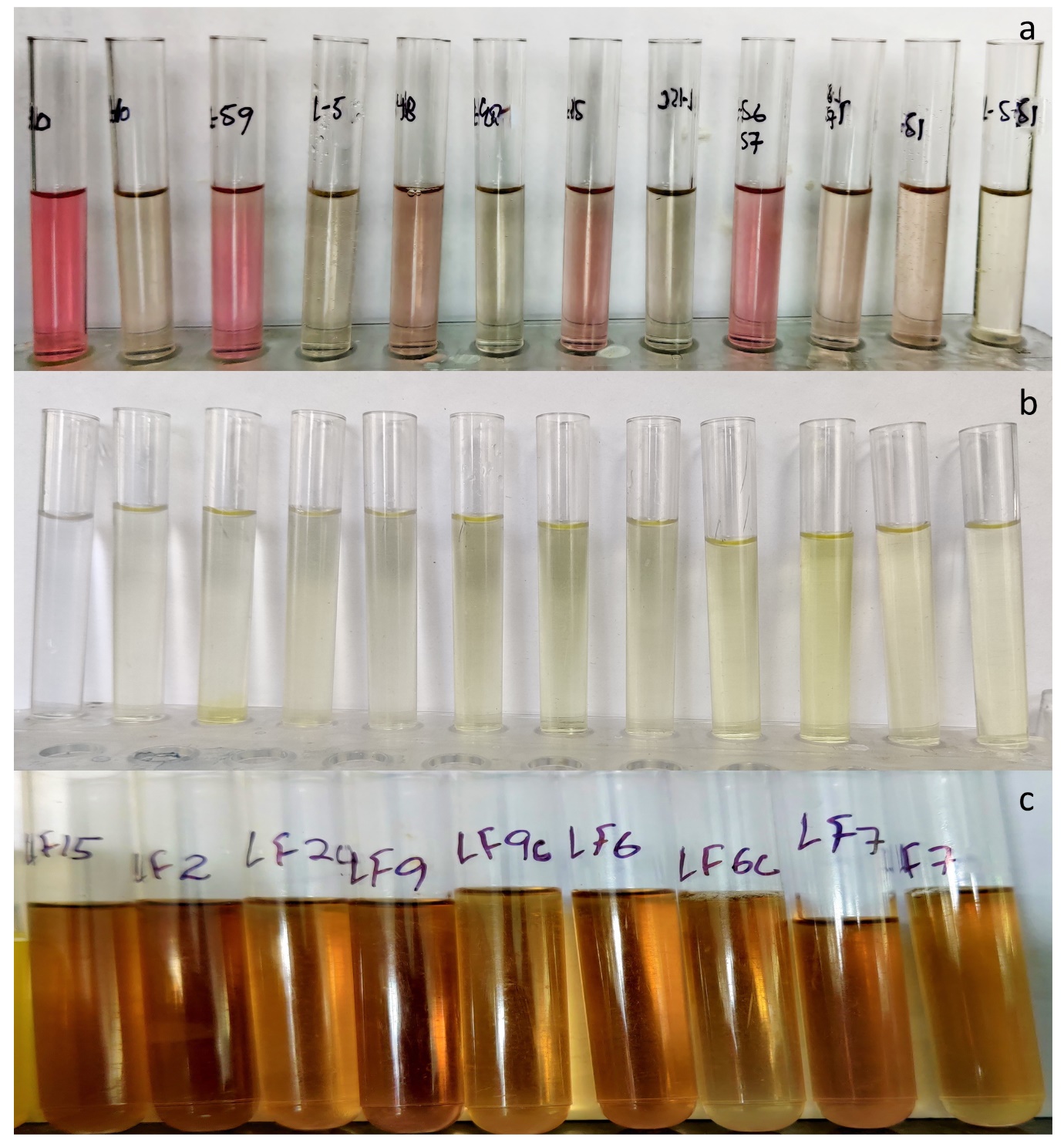


**Supplementary figure S3:** Functional determination of leaf endophytic bacteria and fungi

1. **IAA production b) Phosphate solubilisation c) Ammonia production**


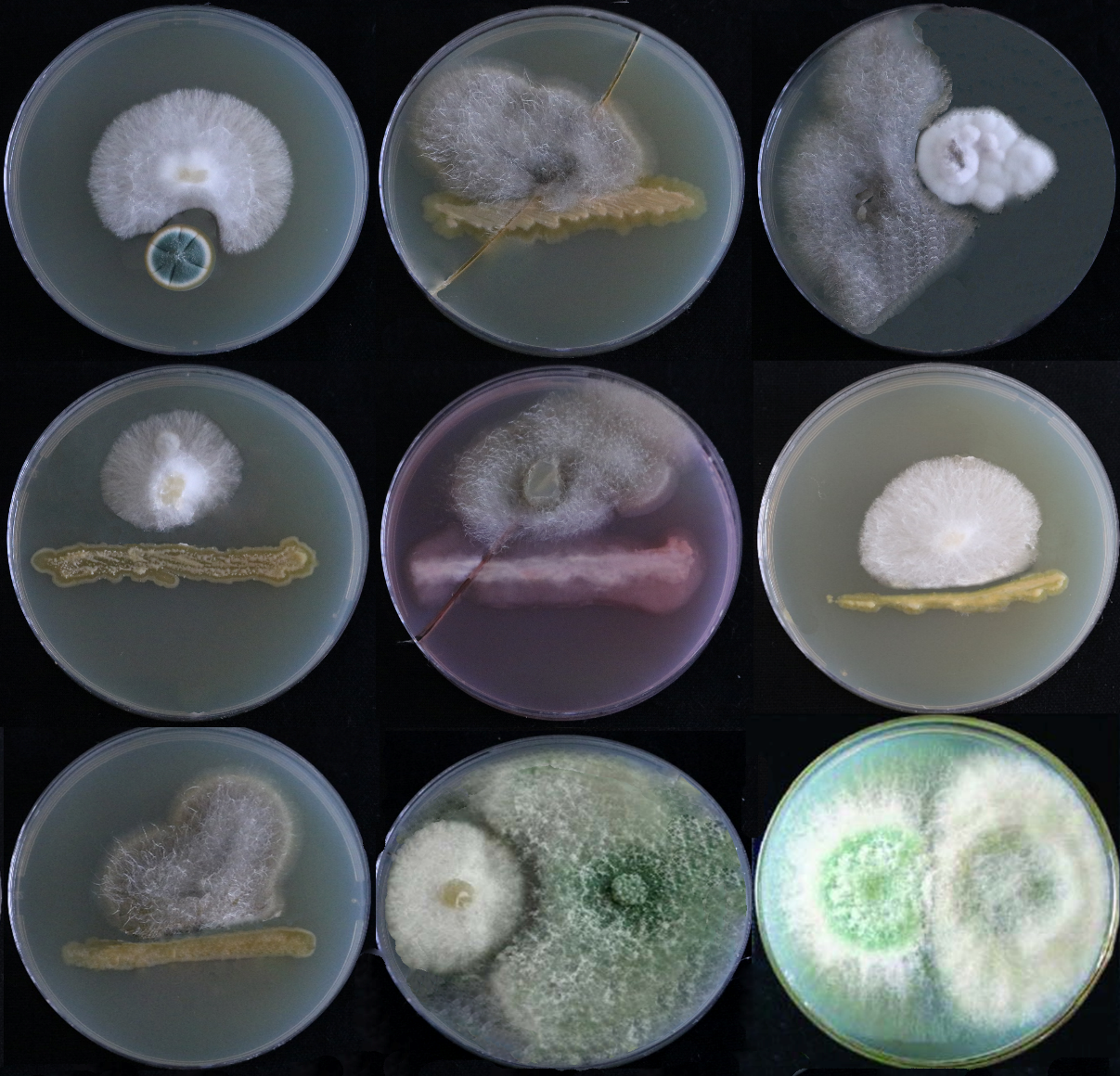


**Supplementary figure S4: Microbial endophytes showing the biocontrol activity**


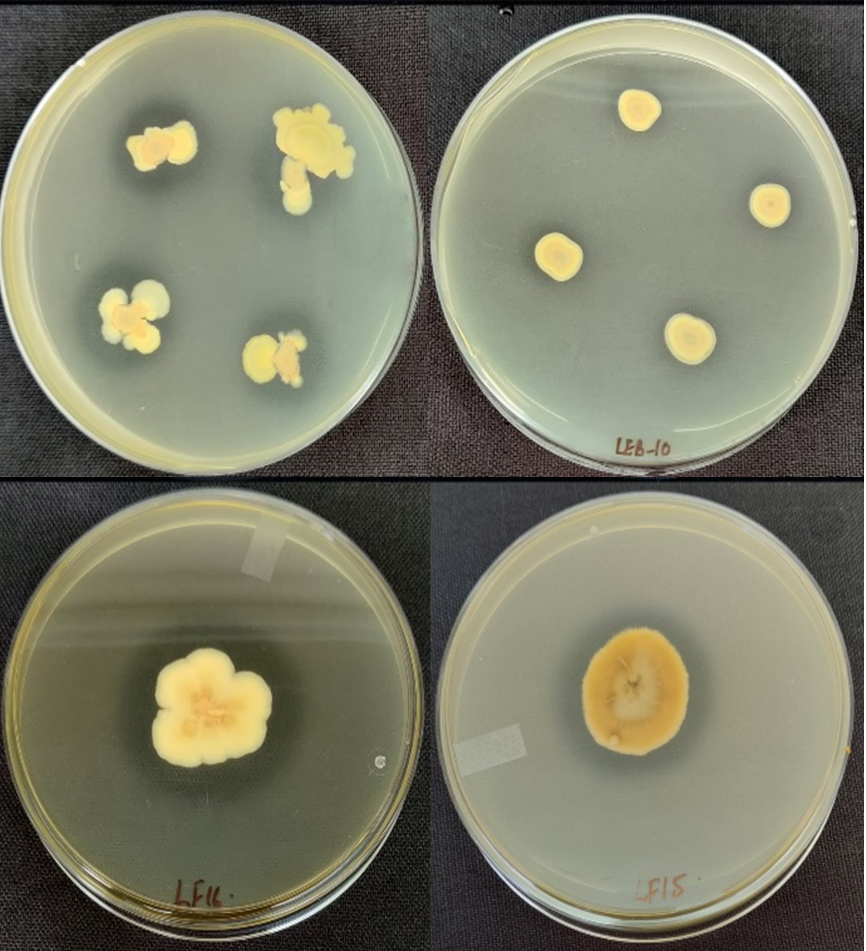


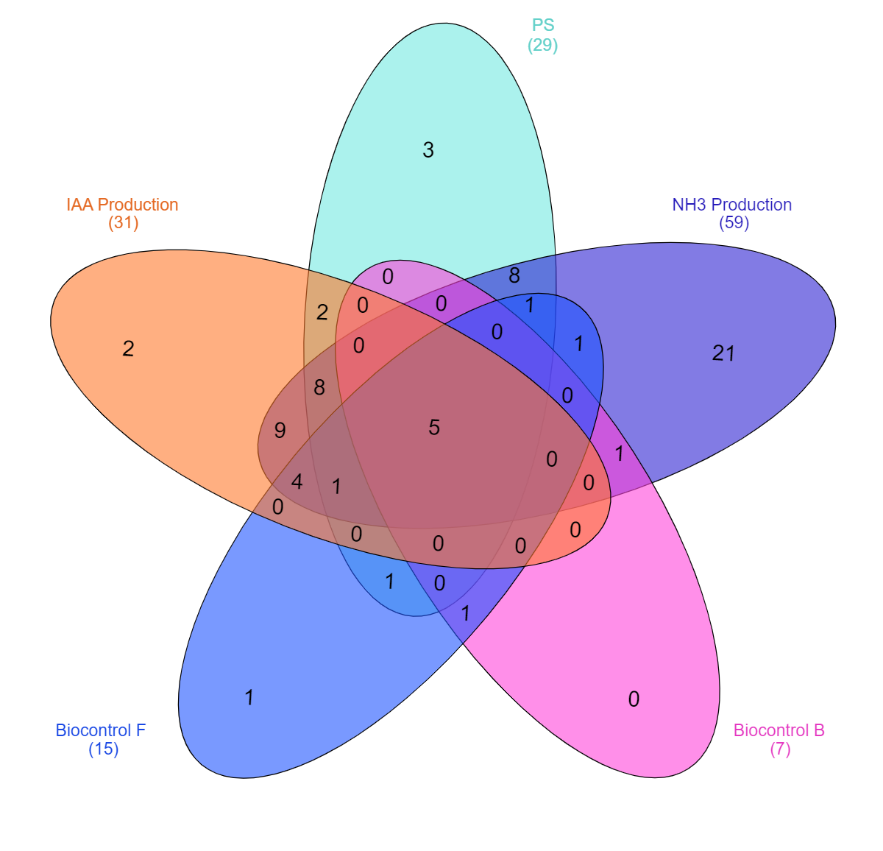
**Supplementary figure S5:** Microbial endophytes showing phosphate solubilisation qualitatively.

**Supplementary fig S6:** Venn diagram showing shared and specific functional traits of obtained endophytes.

**
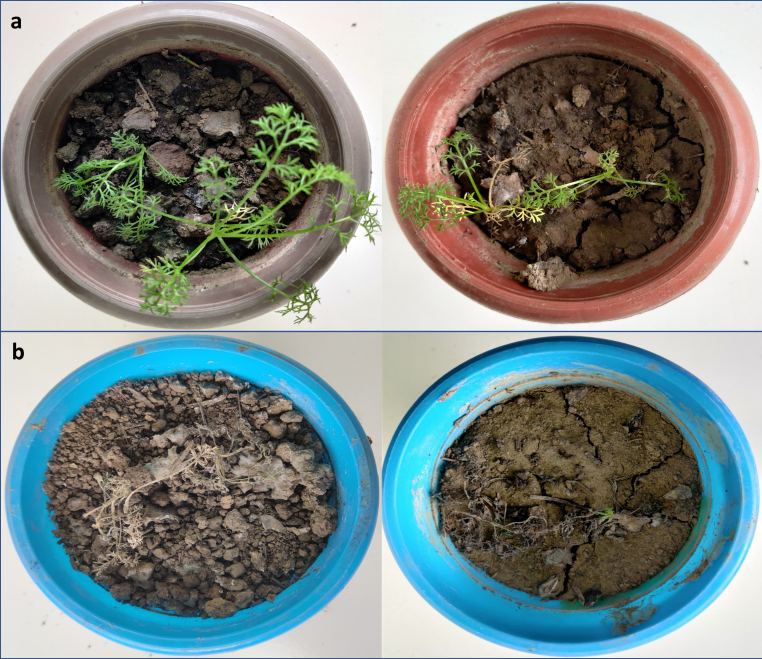
**

**Supplementary figure S7: Effect of microbial consortia showing antagonism against *Botrytis cinerea*. a Treatment of sterilized seeds + inoculum, b Sterilized seeds without inoculum (Control)**

| **Supplementary Table 1: showing diversity and the closest relatives of microbial endophytic OTUs isolated from *Anthemis cotula* based on the 16s and ITS sequence analysed by BLASTn** | | | | |
| --- | --- | --- | --- | --- |
| **Sample_ID** | **Species_Name** | **Accession No.** | **% age similarity** | **Top hit strains in gene bank with accession numbers** |
| L1 | *Erwinia gerundensis* | OL629623 | >99 % | NR_148820.1 *Erwinia gerundensis* |
| L2 | *Pantoea ananatis* | OL629624 | >99 % | KM068005.1 *Pantoea ananatis* |
| L3 | *Pseudomonas chlororaphis* | OL629625 | >99 % | MT102292.1 *Pseudomonas chlororaphis* |
| L4 | *Bacillus firmus* | OL629626 | >99 % | MT457466.1 *Bacillus firmus* |
| L5 | *Bacillus altitudinis* | OL629627 | >99 % | OK335836.1 *Bacillus altitudinis* |
| L6 | *Cellulomonas hominis* | OL629628 | >99 % | KP762562.1 *Cellulomonas hominis* |
| L7 | *Curtobacterium flaccumfaciens* | OL629629 | >99 % | MN826580.1 *Curtobacterium flaccumfaciens* |
| L8 | *Pseudomonas stutzeri* | OL629630 | >99 % | MG905265.1 *Pseudomonas stutzeri* |
| L9 | *Pseudomonas trivialis* | OL629631 | >99 % | MK100814.1 *Pseudomonas trivialis* |
| L10 | *Pseudomonas graminis* | OL629632 | >99 % | KP407118.1 *Pseudomonas graminis* |
| L11 | *Pseudomonas umsongensis* | OL629633 | >99 % | MT628350.1 *Pseudomonas umsongensis* |
| L12 | *Microbacterium schleiferi* | OL629634 | >99 % | MT487643.1 *Microbacterium schleiferi* |
| L13 | *Frigoribacterium faeni* | OL629635 | >99 % | LT718632.1 *Frigoribacterium faeni* |
| L14 | *Okibacterium fritillariae* | OL629636 | >99 % | MT539746.1 *Okibacterium fritillariae* |
| L15 | *Microbacterium testaceum* | OL629637 | >99 % | MT605456.1 *Microbacterium testaceum* |
| L16 | *Pseudomonas viridiflava* | OL629638 | >99 % | MG972916.1 *Pseudomonas viridiflava* |
| L17 | *Xanthomonas retroflexus* | OL629639 | >99 % | MW386578.1 *Xanthomonas retroflexus* |
| L18 | *Pseudomonas putida* | OL629640 | >99 % | HE585987.1 *Pseudomonas putida* |
| L19 | *Chryseobacterium indoltheticum* | OL629641 | >99 % | LN997904.1 *Chryseobacterium indoltheticum* |
| L20 | *Bacillus marisflavi* | OL629642 | >99 % | MT510150.1 *Bacillus marisflavi* |
| L21 | *Frigoribacterium sp* | OL629643 | >99 % | MG860045.1 *Frigoribacterium* sp. |
| L22 | *Rhodococcus trifolii* | OL629644 | >99 % | KY823026.1 *Rhodococcus trifolii* |
| L23 | *Microbacterium proteolyticum* | OL629645 | >99 % | MT214262.1 *Microbacterium proteolyticum* |
| L24 | *Bacillus cereus* | OL629646 | >99 % | MH633904.1 *Bacillus cereus* |
| L25 | *Bacillus amyloliquefaciens* | OL629647 | >99 % | MT542326.1 *Bacillus amyloliquefaciens* |
| L26 | *Bacillus toyonensis* | OL629648 | >99 % | MT605503.1 *Bacillus toyonensis* |
| L27 | *Bacillus safensis* | OL629649 | >99 % | MT379506.1 *Bacillus safensis* |
| L28 | *Bacillus tropicus* | OL629650 | >99 % | MT611943.1 *Bacillus tropicus* |
| L29 | *Bacillus paramycoides* | OL629651 | >99 % | MT875312.1 *Bacillus paramycoides* |
| L30 | *Bacillus pumilus* | OL629652 | >99 % | MN581187.1 *Bacillus pumilus* |
| L31 | *Bacillus sp.* | OL629653 | >99 % | KJ941327.1 *Bacillus* sp. |
| L32 | *Xanthomonas arboricola* | OL629654 | >99 % | MT573402.1 *Xanthomonas arboricola* |
| L33 | *Bacillus albus* | OL629655 | >99 % | MT332155.1 *Bacillus albus* |
| L34 | *Bacillus mojavensis* | OL629656 | >99 % | MN918325.1 *Bacillus mojavensis* |
| L35 | *Rhodococcus qingshengii* | OL629657 | >99 % | MN826591.1 *Rhodococcus qingshengii* |
| L36 | *Bacillus velezensis* | OL629658 | >99 % | KT890344.1 *Bacillus methylotrophicus* |
| L37 | *Sphingobacterium mizutaii* | OL629659 | >99 % | MW261785.1 *Sphingobacterium mizutaii* |
| L38 | *Sphingomonas mali* | OL629660 | >99 % | MK302226.1 *Sphingomonas mali* |
| L39 | *Rhodococcus corynebacterioides* | OL629661 | >99 % | MN826595.1 *Rhodococcus corynebacterioides* |
| L40 | *Brevundimonas sp.* | OL629662 | >99 % | MK729043.1 *Brevundimonas* sp. |
| L41 | *Stenotrophomonas maltophilia* | OL629663 | >99 % | MT199170.1 *Stenotrophomonas maltophilia* |
| L42 | *Paenibacillus amylolyticus* | OL629664 | >99 % | MN865917.1 *Paenibacillus amylolyticus* |
| L43 | *Staphylococcus pasteuri* | OL629665 | >99 % | MH445558.1 *Staphylococcus pasteuri* |
| L44 | *Gordonia hongkongensis* | OL629666 | >99 % | MW828369.1 *Gordonia hongkongensis* |
| L45 | *Bacillus subtilis* | OL629667 | >99 % | MF993345.1 *Bacillus subtilis* |
| L46 | *Bacillus atrophaeus* | OL629668 | >99 % | MN826517.1 *Bacillus atrophaeus* |
| L47 | *Brevibacillus agri* | OL629669 | >99 % | MT422060.1 *Brevibacillus agri* |
| L48 | *Bacillus megaterium* | OL629670 | >99 % | MT394422.1 *Bacillus megaterium* |
| L49 | *Microbacterium sp.* | OL629671 | >99 % | KR906234.1 *Microbacterium* sp. |
| L50 | *Enterobacter sp.* | OL629672 | >99 % | AF283539.2 *Enterobacterium* sp. |
| L51 | *Azospirillum sp.* | OL629673 | >99 % | Z29623.1 *Azospirillum* sp. |
| L52 | *Burkholderia phytofirmans* | OL629674 | >99 % | FJ357149.1 *Burkholderia phytofirmans* |
| L53 | *Bacillus mycoides* | OL629675 | >99 % | LT844639.1 *Bacillus mycoides* |
| L54 | *Taonella mepensis* | OL629676 | >99 % | MT410609.1 *Taonella mepensis* |
| L55 | *Paenibacillus polymyxa* | OM135497 | >99 % | AH011809.2 Bacillus polymyxa |
| L56 | *Pleomorphomonas oryzae* | OL629677 | >99 % | JQ958883.1 *Kocuria rosea* |
| L57 | *Lactobacillus paraplantarum* | OL629679 | >99 % | OK631753.1 *Pleomorphomonas oryzae* |
| L58 | *Microbacterium foliorum* | OL629680 | >99 % | MT622658.1 *Lactobacillus paraplantarum* |
| L59 | *kocuria rosea* | OL629681 | >99 % | MH144232.1 *Microbacterium foliorum* |
| LF1a | *Trichothecium_roseum* | OL636169 | >99 % | MK247947.1 *Trichothecium roseum* |
| LF2 | *Acremonium_sclerotigenum* | OL631261 | >99 % | KX761888.1 *Acremonium sclerotigenum* |
| LF3 | *Aspergillus _oryzae* | OL631262 | >99 % | MF668185.1 *Aspergillus oryzae* |
| LF4 | *Alternaria-alternata* | OL631263 | >99 % | MT646481.1 *Alternaria alternata* |
| LF5 | *Alternaria_tenuissima* | OL631264 | >99 % | MZ379282.1 *Alternaria tenuissima* |
| LF6 | *Trichoderma_viride* | OL631265 | >99 % | JF440620.1 *Trichoderma viride* |
| LF7 | *Alternaria-infectoria* | OL631266 | >99 % | JF440601.1 *Alternaria infectoria* |
| LF8 | *Aspergillus_tubingensis* | OL631267 | >99 % | HQ262500.1 *Aspergillus tubingensis* |
| LF9 | *Perpureocillium_lilacium* | OL631268 | >99 % | MT606206.1 *Purpureocillium* sp. |
| LF10 | *Myrothecium_sp* | OL631269 | >99 % | MN202732.1 *Myrothecium* sp. |
| LF11 | *Aspergillus_niger* | OL631270 | >99 % | MT628904.1 *Aspergillus niger* |
| LF12 | *Penicillium-commune* | OL631271 | >99 % | FJ499454.1 *Penicillium commune* |
| LF13 | *Nigrospora_oryzae* | OL631272 | >99 % | MN341492.1 *Nigrospora oryzae* |
| LF14 | *cladosporium_perangustum* | OL631273 | >99 % | MT645919.1 *Cladosporium perangustum* |
| LF15 | *Cladosporium allicinum* | OL631274 | >99 % | MG646341.1 *Cladosporium allicinum* |
| LF16 | *Phoma_sp* | OL631275 | >99 % | MK299419.1 *Phoma* sp. |
| LF17 | *Rhizopus_microsporum* | OL631276 | >99 % | KT737216.1 *Rhizopus microsporus* |
| LF18 | *Cladosporium-sphaerospermum* | OL631277 | >99 % | MT645920.1 *Cladosporium sphaerospermum* |
| LF19 | *Mortierella_sp* | OL631278 | >99 % | MZ768910.1 *Mortierella* sp. |
| LF20 | *Pestalotiopsis_sp* | OL631279 | >99 % | JF304629.1 *Pestalotiopsis* sp. |

| **Supplementary Table 2: Diversity of endophytes and their taxonomic information.** | | | | | |
| --- | --- | --- | --- | --- | --- |
| **Phylum** | **Class** | **Order** | **Family** | **Genus** | **Species** |
| Proteobacteria | Gama-proteobacteria | Enterobacteriales | Erwinaceae | *Erwinia* | *gerundensis* (6) |
|  |  |  |  | *Pantoea* | *ananatis* (7) |
|  |  | Pseudomonadales | Pseudomonadaceae | *Pseudomonas* | *chlororaphis* (14) |
|  |  |  |  | *Pseudomonas* | *virdiflava* (11) |
|  |  |  |  | *Pseudomonas* | *trivialis* (15) |
|  |  |  |  | *Pseudomonas* | *graminis* (9) |
|  |  |  |  | *Pseudomonas* | *umsongensis* (11) |
|  |  |  |  | *Pseudomonas* | *stutzeri* (11) |
|  |  |  |  | *Pseudomonas* | *putida* (19) |
|  |  |  |  | *Pleomorphomonas* | *oryzae* (11) |
|  |  | Xanthomonadales |  | *Xanthomonas* | *retrophelexus* (8) |
|  |  |  |  | *Xanthomonas* | *arbicola* (4) |
|  |  | Enterobacterales | Enterobacteraceae | *Enterobacter* | sp. (10) |
|  |  |  |  | *Stenotrophomonas* | *maltophila* (7) |
|  |  | Caulobacterales | Caulobacteraceae | *Brevundimonas* | sp. (12) |
|  | Alpha-proteobacteria | Sphingomonadales | Sphingomonadaceae | *Sphingomonas* | *mali* (9) |
|  |  | Rhodospirilales | Azospirillaceae | *Azospirillum* | sp. (8) |
|  |  |  | Rhodospiraceae | *Taonella* | *mepensis* (4) |
|  | Beta-proteobacteria | Burkholderiales | Burkholderaceae | *Burkholderia* | *phytofirmans* (11) |
| Firmicutes | Bacilli | Bacillales | Bacillaceae | *Bacillus* | *firmus* (26) |
|  |  |  |  | *Bacillus* | *altitudinis* (5) |
|  |  |  |  | *Bacillus* | *marisflavi* (22) |
|  |  |  |  | *Bacillus* | *cereus* (28) |
|  |  |  |  | *Bacillus* | *amyloliquifaciens* (22) |
|  |  |  |  | *Bacillus* | *safensis* (25) |
|  |  |  |  | *Bacillus* | *paramycoides* (22) |
|  |  |  |  | *Bacillus* | *tropicus* (23) |
|  |  |  |  | *Bacillus* | *pumilus* (21) |
|  |  |  |  | *Bacillus* | *mycoides* (4) |
|  |  |  |  | *Bacillus* | *albus* (20) |
|  |  |  |  | *Bacillus* | *mojavensis* (22) |
|  |  |  |  | *Bacillus* | *toyonensis* (24) |
|  |  |  |  | *Bacillus* | *atrophies* (14) |
|  |  |  |  | *Bacillus* | *megaterium* (20) |
|  |  |  |  | *Bacillus* | *subtilis* (14) |
|  |  |  |  | *Bacillus* | *velezensis* (28) |
|  |  |  |  | *Bacillus* | sp. (23) |
|  |  |  | Staphylocopcaceae | *Staphylococcus* | *pasteuri* (12) |
|  |  | Paenibacillales | Paenibacillaceae | *Paenibacillus* | *amylolyticus* (16) |
|  |  |  |  | *Brevibacillus* | *agri* (12) |
|  |  |  |  | *Bacillus* | *polymyxa* (26) |
|  |  | Lactobacillales | Lactobacillaceae | *Lactobacillus* | *paraplantarum* (10) |
| Actinobacteria | Actinobacteria  Flavobacteria | Micrococcales | Microbacteriaceae | *Microbacterium* | *schleiferi* (22) |
|  |  |  |  | *Frigoribacterium* | *faeni* (14) |
|  |  |  |  | *Microbacterium* | *testaceum* (10) |
|  |  |  |  | *Okibacterium* | *fritillariae* (7) |
|  |  |  |  | *Frigoribacterium* | sp. (4) |
|  |  |  |  | *Microbacterium* | *protolyticum* (14) |
|  |  |  |  | *Microbacterium* | sp. (19) |
|  |  |  |  | *Microbacterium* | *foliorum* (11) |
|  |  |  |  | *Curtobacterium* | *flaccumfaciens* (14) |
|  |  |  |  | *kocuria* | *rosea* (8) |
|  |  |  | Cellulomonadaceae | *Cellulomonas* | *hominis* (5) |
|  |  | Mycobacteriales | Norcadiaceae | *Rhodococcus* | *trifolii* (14) |
|  |  |  |  | *Rhodococcus* | *corynebacteroides* (8) |
|  |  |  |  | *Rhodococcus* | *qingshengii* (6) |
|  |  |  | Gordoniaceae | *Gordonia* | *hongkongensis* (8) |
| Bacteriodetes |  | Flavobacterales | Flavobacteriaceae | *Sphingobacterium* | *mizutaii* (7) |
|  |  |  |  | *Chyseobacterium* | *indolthecium* (3) |
| Division Ascomycota | Sordariomycetes | Hypocreales |  | *Trichothecium* | *roseum* (7) |
|  |  |  | Hypocreaceae | *Acremonium* | *sclerotigenum* (3) |
|  |  |  |  | *Trichoderma* | *viride* (9) |
|  |  |  | Stachybotryaceae | *Myrothecium* | *sp.* (6) |
|  |  |  | Ophiocordycipitaceae | *Perpureocillium* | *lilacinum* (1) |
|  |  | Trichosphaeriales | Trichosphaeriaceae | *Nigrospora* | *oryzae* (6) |
|  |  | Xylariales | Sporocadaceae | *Pestalotiopsis* | sp. (2) |
|  | Dothideomycetes | Pleosporales | Pleosporaceae | *Alternaria* | *alternate* (7) |
|  |  |  |  | *Alternaria* | *tenuissima* (12) |
|  |  |  |  | *Alternaria* | *infectoria* (5) |
|  |  | Capnodiales | Didymellaceae | *Phoma* | sp. (3) |
|  |  |  |  | *cladosporium* | *allicinum* (1) |
|  |  | Eurotiales |  | *cladosporium* | *perangustum* (6) |
|  | Eurotiomycetes | Mortierellales | Trichocomaceae | *Aspergillus* | *oryzae* (4) |
|  |  |  |  | *Penicillium* | *commune* (4) |
|  |  |  |  | *Aspergillus* | *tubingensis* (7) |
|  |  |  |  | *Aspergillus* | *niger* (7) |
| Division Zygomycota | Zygomycetes | Capnodiales | Mortierellaceae | *Mortierella* | *sp.* (2) |
|  |  | Mucorales | Mucoraceae | *Rhizopus* | *microspora* (4) |
